# Speaking Up About Patient Safety, Withholding Voice and Safety Climate in Clinical Settings: a Cross-Sectional Study Among Ibero-American Healthcare Students

**DOI:** 10.3389/ijph.2024.1607406

**Published:** 2024-07-01

**Authors:** Irene Carrillo, Piedad Serpa, Edgar Landa-Ramírez, Mercedes Guilabert, Yesenia Gómez-Ayala, Adriana López-Pineda, José Joaquín Mira

**Affiliations:** ^1^ Department of Health Psychology, Miguel Hernández University of Elche, Elche, Spain; ^2^ Department of Clinical Management and Patient Safety, School of Medicine, Universidad de Santander, Bucaramanga, Colombia; ^3^ Facultad de Psicología, National Autonomous University of Mexico, Mexico City, Mexico; ^4^ Programa de Psicología Urgencias, Hospital General Dr. Manuel Gea Gonzalez, Mexico City, Mexico; ^5^ Clinical Medicine Department, School of Medicine, Miguel Hernández University of Elche, Elche, Spain; ^6^ Research Network on Chronicity, Primary Care and Health Promotion (RICAPPS), Barcelona, Spain; ^7^ ATENEA Research Group, Fundación para el Fomento de la Investigación Sanitaria y Biomédica de la Comunitat Valenciana (FISABIO), Valencia, Spain; ^8^ Alicante-Sant Joan Health District, Alicante, Spain

**Keywords:** speaking up, patient safety, psychological safety, students, survey

## Abstract

**Objectives:**

To explore speaking up behaviours, barriers to openly expressing patient safety concerns, and perceived psychological safety climate in the clinical setting in which healthcare trainees from Ibero-America were receiving their practical training.

**Methods:**

Cross-sectional survey of healthcare trainees from Colombia, Mexico, and Spain (N = 1,152). Before the field study, the Speaking Up About Patient Safety Questionnaire (SUPS-Q) was translated into Spanish and assessed for face validity. A confirmatory factor analysis was conducted to establish the construct validity of the instrument, and the reliability was assessed. The SUPS-Q was used to evaluate voice behaviours and the perceived psychological safety climate among Ibero-American trainees. Descriptive and frequency analyses, tests for contrasting means and proportions, and logistic regression analyses were performed.

**Results:**

Seven hundred and seventy-one trainees had experience in clinical settings. In the previous month, 88.3% had experienced patient safety concerns, and 68.9% had prevented a colleague from making an error. More than a third had remained silent in a risky situation. Perceiving concerns, being male or nursing student, and higher scores on the encouraging environment scale were associated with speaking up.

**Conclusion:**

Patient safety concerns were frequent among Ibero-American healthcare trainees and often silenced by personal and cultural barriers. Training in speaking up and fostering safe interprofessional spaces is crucial.

## Introduction

Patient safety education for healthcare students has been recognised by national and international accreditation bodies and agencies as a priority [1] because of the frequency with which patients suffer harm derived from healthcare [2]. Different approaches to working on patient safety competencies [3, 4] and tools for their implementation and assessment in curricula have been developed in response to this need [5, 6].

Healthcare students and residents (hereafter “trainees”) are inevitably involved in patient safety incidents, either as subjects or observers, making them valuable sources of information and agents of change [7]. Between 76% and 92% of medical students acknowledge witnessing an error during their clerkships, whereas 18%–25% admit to being responsible for an error [8–10]. The likelihood of making an error increase during residency [11].

Despite the countless guidelines and proposals developed to incorporate patient safety into undergraduate and graduate healthcare education levels [1, 3, 4], their widespread implementation remains a challenging issue of varying intensity, depending on the country and speciality [12, 13]. In Europe, several studies show that the patient safety subject as an independent entity is the exception in nursing and medical curricula [14, 15]. In low- and middle-income countries, while the adoption of patient safety curricula is under consideration or planning, implementation remains challenging [16]. There is hardly any literature that analyses the differences in patient safety training of healthcare students between Spanish-speaking countries in the Americas and Europe. However, the few studies available suggest that, since patient safety education is still far from being homogeneous at the national level, differences between countries may be due to multiple factors [14–16]. In a study with medical and nursing students from different Ibero-American countries, Colombian students showed better patient safety attitudes and knowledge than Spanish students [17]. However, these results cannot be generalised. Possible differences between countries may be due to variations in curricula. However, the Americas and Europe generally include clinical internships from the third or fourth year onward. From a cultural point of view, in countries such as Colombia and Mexico, traditional medicine practices are still present in rural and indigenous areas, which poses an additional challenge in guaranteeing patient safety [18, 19], compared to Spain, where these practices are less widespread. Formal patient safety training is still pending in most Ibero-American countries, although most have reported isolated institutional initiatives [18, 20].

Studies show that trainees can be trained to effectively detect and report adverse events and contribute to improving patient safety and quality of care [21]. To this end, they must be allowed to express their concerns and suggestions in a climate of trust and respect. Psychological safety is an interpersonal construct that refers to the consensus in individuals’ perceptions of the consequences of taking interpersonal risks (like speaking up or asking for help) in their work environment [22] or to the belief that one can express oneself without fear of criticism from others or negative consequences to self-image, status, or career [23]. In healthcare contexts, psychological safety contributes to patient safety and quality of care through speaking up behaviours [24].

Speaking up has been defined as assertive communication of patient safety concerns through information, questions, or opinions where immediate action is needed to avoid patient harm [25]. Despite the proven positive effects of this behaviour, multiple factors lead to individuals’ withholding voice. Some of these barriers to speaking up behaviour are hierarchy, perceived lack of knowledge, dominant or shy personalities, authoritarian leadership, and fear of unpredictable or negative reactions from others [26, 27].

To the authors’ knowledge, the frequency with which future generations of healthcare professionals in Ibero-America engage in speaking up and withholding voice behaviours and their perception of the work climate and barriers to psychological safety have not been studied. The aim of this study was twofold: on the one hand, to adapt and validate the Speaking Up About Patient Safety Questionnaire (SUPS-Q) [28] in Spanish native healthcare trainees from Ibero-America and, on the other hand, to explore their speaking up behaviours, barriers to openly expressing their patient safety concerns, and perceived psychological safety climate in the clinical setting in which they were receiving their practical training.

## Methods

### Study Population and Procedure

A cross-sectional survey-based study among healthcare trainees doing clinical internships at one Ibero-American academic teaching healthcare centre was conducted. Specifically, the survey was disseminated through a convenience sample from academic and healthcare institutions in Colombia, Mexico, and Spain. However, mobility conditions during training were considered, so that trainees from other Ibero-American countries who were in one of the three dissemination countries at the time of the survey were also included. Given the variability in the timing of internship periods in the curricula between countries, recruitment of participants was not based on a specific term in the study programme. To determine if respondents had completed an internship in a health or socio-health centre during their training, they were asked to indicate this information in the survey.

The survey was disseminated by the collaborating professors to a convenience subgroup of students from participant universities. An online invitation message with information about the study’s purpose and the voluntary and anonymous nature of participation was sent to the trainees. They were informed that they were agreeing to participate in the study by responding to the survey. The online survey was open from April 2021 to February 2022. Three reminders were scheduled to increase the response rate. There was no sample size estimation as the study had an explorative character.

### Survey Instrument

The SUPS-Q, originally developed and validated by Richard et al. [28] in healthcare professionals in Switzerland, was adapted to Spanish. This questionnaire was designed to assess healthcare workers’ perceived patient safety concerns, past speaking up behaviours, perceived barriers to speaking up, evaluations of the speaking up climate at their workplace, and their anticipated speaking up behaviour. Three researchers independently carried out the back-translation of the questionnaire. Discrepancies were resolved through joint discussion and consensus building. Respecting the structure of the original questionnaire, the authors agreed on some modifications in the wording and content of the items, as well as the incorporation of new questions considered relevant to the cultural context of the application of the instrument in this study. The authors approved the final translation of the questionnaire into Spanish. Five undergraduate students from each participating country assessed the face validity of the instrument. Overall, the students found the questionnaire content easy to understand and relevant. Minor adjustments were made to the wording of the items based on the students’ suggestions.

Respecting the structure of the SUPS-Q, the instrument consisted of three behaviour scales (perceived concerns -PC-, withholding voice -WV-, and speaking up -SU-), three climate sub-scales (psychological safety for speaking up -PSS-, encouraging environment for speaking up -EES-, and resignation toward speaking up -RES-), a predefined list of barriers to speaking up and a vignette describing a generic situation requiring speaking up.

Speaking up behaviours were assessed with 11 items, addressing the frequency of perceived safety concerns due to errors and non-compliance rules (PC1-3), withholding voice behaviours (WV1-4) (choose not to speak up in specified situations), and speaking up behaviours (SU1-4) over the past 4 weeks. A 5-point Likert scale was used for PC, WV, and SU, whose response options were “never” (0 times in the last 4 weeks), “rarely” (1-2 times), “sometimes” (3-5 times), “often” (6–10 times), and “very often” (more than 10 times in the last 4 weeks). Thus, higher mean scale values indicated higher frequencies of past speaking up and withholding voice behaviours.

Speak up-related climate was assessed with 10 items that explore whether healthcare trainees perceive their environment, colleagues, and supervisors/mentors as supportive to speaking up (EES1-3), their level of resignation with speaking up (RES1-2), and psychological safety (PSS1-5). The answers to the EES, RES and PSS items were coded in a 7-point Likert scale from “strongly disagree” to “strongly agree,” including a “not applicable” response option.

A predefined list of eight factors (PB1-8) and a 4-point Likert scale were used to identify those aspects that trainees perceived as barriers to bringing up patient safety concerns (from “not at all” to “completely”).

A clinical vignette describing a hypothetical situation was used to assess trainees anticipated speaking up behaviours. The vignette reads “You are observing how a specific procedure is applied to a patient. A professional is about to examine the surgical wound of the patient. However, he/she does not put on gloves and has not hygienised their hands.” Trainees were asked to complete four questions assessing the realism of the situation, the potential for patient harm, their discomfort with and likelihood of speaking up. These questions each used a 7-point Likert response scale with specifically labelled poles.

Additionally, the following sociodemographic variables were recorded: sex, age, profile, or healthcare discipline (nursing, medicine, psychology or other), year of beginning of studies and performance of an internship in a health or social care centre.

### Statistical Analysis

Descriptive and frequency analyses were performed for items and subscales. Comparative analyses were performed according to the level of training (with vs. without practice), the profile or health discipline (nursing vs. medicine) and the sex (female vs. male) of the respondents. Participants who indicated that they had no practice at the time of the survey were only considered as a control group for the comparative analysis according to the level of training. These participants were discarded for the rest of the analysis as they did not meet the inclusion criteria. Non-parametric Mann-Whitney U and Chi-Square tests were used to contrast means and proportions, respectively. The probability of superiority (PS) for the Mann-Whitney U test (0.53 very small, 0.56 small, 0.64 medium, 0.71 large, 0.80 very large and 0.92 huge) [29] and Cramér’s V for the Chi-Square test (≤0.10 negligible, >0.10 to ≤0.20 weak, >0.20 to ≤0.40 moderate, >0.40 to ≤0.60 relatively strong, >0.60 to ≤0.80 strong, and >0.80 very strong) [30] were used to calculate the effect size. In both cases, the value ranges from 0-1, indicating a larger effect size as the values approach 1.

A binary logistic regression using the enter method was conducted to describe the association between two response variables (likelihood of speaking up -SU- and withholding voice behaviours -WV-) and the following independent variables: sex, age, student profile, PC, PB, PSS, EES and RES. For these analyses, the dependent variables (behaviour scales SU and WV) were dichotomised, where 0 = absence of SU and WV behaviours in the last 4 weeks (responses “never” or “rarely”), and 1 = presence of at least one SU or WV behaviour 3 or more times in the last month.

Regarding instrument reliability, Cronbach’s alpha and the McDonald’s omega coefficients were calculated to assessed internal consistency of scales with values >0.7 [31] and >0.6 [32] indicating acceptable consistency, respectively.

A confirmatory factor analysis (CFA) was conducted using maximum likelihood estimation methods to test the defined six-scale structure of the SUPS-Q to which the perceived barriers’ items were added. Model fit was assessed using Chi-square statistic (χ^2^), Chi-square divided by degrees of freedom (χ^2^/df) (acceptable fit ≤5, good fit <2), Comparative Fit Index (CFI) and Tucker Lewis Index (TLI) (acceptable fit 0.90-0.95, good fit ≥0.95), Root Mean Square Error of Approximation (RMSEA) (good fit ≤0.05), Standardized Root Mean Squared Residual (SRMR) (acceptable fit <0.10, good fit ≤0.05), Adjusted Goodness-of-Fit Index (AGFI) (acceptable fit 0.85–0.90, good fit >0.90) [33, 34].

Data analyses were performed with IBM SPSS Statistics and IBM SPSS Amos 28.0.0.

### Ethical Approval

The study was approved by the Research Integrity and Ethics Committee of the Miguel Hernández University of Elche (record code: 2021/52945) and the Research Commission of the University Hospital San Juan de Alicante (27 April 2021), Spain.

## Results

Using a convenience sample, 1,152 students from healthcare disciplines in Ibero-American countries completed the questionnaire. Of these, 771 (66.9%) had done internships in clinical or socio-health contexts. Further sample characteristics are provided in Table 1.

**TABLE 1:** Characteristics of the study sample (Colombia, Mexico, and Spain, 2021–2022).

	Without practice (*n* = 381)	With practice (*n* = 771)	Total (*n* = 1152)
Sex, n (%)			
Female	266 (70.1)	565 (73.3)	832 (72.2)
Male	114 (29.9)	206 (26.7)	320 (27.8)
Age, M (SD)	20.7 (4.1)	25.0 (5.5)	23.6 (5.5)
17–21 years, n (%)	308 (80.8)	206 (26.7)	514 (44.6)
22–24 years, n (%)	40 (10.5)	273 (35.4)	313 (27.2)
25–30 years, n (%)	20 (5.2)	198 (25.7)	218 (18.9)
31–40 years, n (%)	8 (2.1)	73 (9.5)	81 (7.0)
> 40 years, n (%)	5 (1.3)	21 (2.7)	26 (2.3)
Country			
Colombia	33 (8.7)	294 (38.1)	327 (28.4)
Mexico	322 (84.5)	264 (34.2)	586 (50.9)
Spain	6 (1.6)	101 (13.1)	107 (9.3)
Other	20 (5.2)	112 (14.5)	132 (11.5)
Profile or study discipline			
Nursing student or nurse resident	43 (11.3)	305 (39.6)	348 (30.2)
Medical student or medical resident	304 (79.8)	374 (48.5)	678 (58.9)
Psychology student or psychologist resident	34 (8.9)	92 (11.9)	126 (10.9)

### Confirmatory Factor Analysis and Reliability of the Instrument

The results of the confirmatory factor analysis conducted using the data from the students with clinical experience (*n* = 771) revealed an acceptable to good model fit. χ^2^/df (2.26), TLI (0.95) and SRMR (0.09) showed acceptable fit, while the CFI (0.96), AGFI (0.92), and RMSEA (0.04) values indicated a good fit. The standardized coefficients for the seven-factor model of SUPS-Q are represented in Supplementary Material S1. Cronbach’s alpha and McDonald’s omega for the behaviour and climate scales ranged from 0.74 to 0.87, indicating acceptable to good internal consistencies (Tables 2–4).

**TABLE 2:** Frequencies of reporting perceived concerns (PC), withholding voice (WV), speaking up behaviours (SU) and perceived barriers (PB) to speaking up, % (n)^a^ (Colombia, Mexico, and Spain, 2021–2022).

**Perceived concerns** *(Cronbach’s α = 0.74; McDonald’s ω = 0.74)* *Over the last 4 weeks, how often…*	**Never**	**Rarely**	**Sometimes**	**Often**	**Very often**	**At least once** ^**b**^
PC1 … have you had specific concerns about patient safety?	11.7 (90)	24.9 (192)	32.6 (251)	21.1 (163)	9.7 (75)	88.3 (681)
PC2 … have you observed a failure/error that, if uncaptured timely, could be harmful to patients?	37.5 (289)	31.3 (241)	22.7 (175)	6.7 (52)	1.8 (14)	62.5 (482)
PC3 … have you noticed that a professional in the unit or service in which you are training has not followed important patient safety rules or standards?	36.7 (283)	30.5 (235)	18.8 (145)	9.2 (71)	4.8 (37)	63.3 (488)
**Withholding voice** *(Cronbach’s α = 0.79; McDonald’s ω = 0.80)* *Over the last 4 weeks, how often…*	**Never**	**Rarely**	**Sometimes**	**Often**	**Very often**	**At least once** ^**b**^
WV1 … have you kept ideas that could improve patient safety in the unit or department where you are training?	35.7 (275)	29.4 (227)	19.6 (151)	11.2 (86)	4.2 (32)	64.3 (496)
WV2 … have you chosen to remain silent and not say anything when witnessing a risky situation for a patient?	62.1 (479)	21.4 (165)	9.7 (75)	5.2 (40)	1.6 (12)	37.9 (292)
WV3 … have you remained silent despite having information that could have prevented a safety incident in the unit or service where you are training?	72.2 (557)	17.3 (133)	7.4 (57)	1.9 (15)	1.2 (9)	27.8 (214)
WV4 … have you avoided warning any professional in the unit or service where you are training that they were overlooking important patient safety rules/standards?	59.5 (459)	22.6 (174)	12.6 (97)	4.0 (31)	1.3 (10)	40.5 (321)
**Speaking up** *(Cronbach’s α = 0.85; McDonald’s ω = 0.85)* *Over the last 4 weeks, how often…*	**Never**	**Rarely**	**Sometimes**	**Often**	**Very often**	**At least once** ^**b**^
SU1 … have you explicitly shared your patient safety concerns with your supervisor, mentor, or other professionals on the unit or service?	21.1 (163)	25.0 (193)	23.3 (180)	21.9 (169)	8.6 (66)	78.9 (608)
SU2 … have you helped prevent another professional from making an error that could have caused harm to a patient?	31.1 (240)	29.1 (224)	24.4 (188)	12.7 (98)	2.7 (21)	68.9 (531)
SU3 … have you warned a professional in the unit or department where you are training that they were overlooking important patient safety rules/standards?	40.7 (314)	30.6 (236)	18.2 (140)	8.3 (64)	2.2 (17)	59.3 (457)
SU4 … have you prevented an incident from occurring by making concrete proposals to increase patient safety?	39.7 (306)	29.2 (225)	21.0 (162)	8.3 (64)	1.8 (14)	60.3 (465)
**Perceived barriers** *(Cronbach’s α = 0.86; McDonald’s ω = 0.86)*	**Not at all**	**Partially**	**Moderately**	**Completely**		
PB1. It is not clear that the situation represents a risk for a patient	34.2 (264)	48.4 (373)	12.5 (96)	4.9 (38)		
PB2. Fear of a negative reaction from professionals or teachers/mentors	30.4 (234)	30.2 (233)	23.1 (178)	16.3 (126)		
PB3. The presence of patients at that moment	30.5 (235)	38.5 (297)	20.4 (157)	10.6 (82)		
PB4. Doubts about how best to say it	24.4 (188)	42.3 (326)	23.7 (183)	9.6 (74)		
PB5. Feel that one lacks sufficient social and communication skills to talk about it	38.1 (294)	39.2 (302)	16.1 (124)	6.6 (51)		
PB6. The unpredictable reaction of the service or unit manager	31.4 (242)	36.6 (282)	18.5 (143)	13.5 (104)		
PB7. Lack of self-confidence to discuss these issues with mentors or professionals	39.9 (308)	36.4 (281)	16.2 (125)	7.4 (57)		
PB8. Believe that talking about these issues will negatively impact my current and future involvement with the centre	34.1 (263)	36.2 (279)	16.1 (124)	23.6 (105)		

N = 771.

^a^ For perceived concerns (PC), withholding voice (WV), speaking up (SU) categories were presented as: “never” (0 times in the last 4 weeks), “rarely” (1-2 times in the last 4 weeks), “sometimes” (3–5 times in the last 4 weeks), “often” (6–10 times in the last 4 weeks), and “very often” (more than 10 times in the last 4 weeks).

^b^ The sum of the categories “rarely,” “sometimes,” “often” and “very often.”

**TABLE 3:** Mean (SD) responses to perceived concerns (PC), withholding voice (WV), speaking up behaviours (SU) and perceived barriers (PB) to speaking up by student profile and sex^a^ ^,^ ^b^ (Colombia, Mexico, and Spain, 2021–2022).

**Perceived concerns** *(Cronbach’s α = 0.74; McDonald’s ω = 0.74)* *Over the last 4 weeks, how often…*	**Total^c^ (*N* = 771)**	**Female (*n* = 565)**	**Male (*n* = 206)**	***p***	**PS**	**Nursing (*n* = 305)**	**Medicine (*n* = 374)**	***p***	**PS**
PC1 … have you had specific concerns about patient safety?	1.9 (1.1)	1.9 (1.2)	2.1 (1.1)	0.003	0.43	2.0 (1.2)	2.0 (1.1)	0.888	0.50
PC2 … have you observed a failure/error that, if uncaptured timely, could be harmful to patients?	1.0 (1.0)	1.0 (1.0)	1.2 (1.0)	0.046	0.46	1.0 (1.0)	1.1 (1.1)	0.391	0.48
PC3 … have you noticed that a professional in the unit or service in which you are training has not followed important patient safety rules or standards?	1.1 (1.2)	1.1 (1.2)	1.2 (1.1)	0.214	0.47	1.2 (1.2)	1.1 (1.1)	0.456	0.48
Total perceived concerns^d^	7.2 (4.7)	7.0 (4.7)	7.9 (4.6)	0.007	0.44	7.3 (4.6)	7.3 (4.8)	0.976	0.50
**Withholding voice** *(Cronbach’s α = 0.79; McDonald’s ω = 0.80)* *Over the last 4 weeks, how often…*	**Total** ^c^ **(*N* = 771)**	**Female (*n* = 565)**	**Male (*n* = 206)**	***p***	**PS**	**Nursing (*n* = 305)**	**Medicine (*n* = 374)**	***p***	**PS**
WV1 … have you kept ideas that could improve patient safety in the unit or department where you are training?	1.2 (1.2)	1.2 (1.2)	1.3 (1.1)	0.174	0.47	1.1 (1.2)	1.2 (1.2)	0.217	0.47
WV2 … have you chosen to remain silent and not say anything when witnessing a risky situation for a patient?	0.6 (1.0)	0.6 (0.9)	0.7 (1.0)	0.087	0.47	0.5 (0.9)	0.7 (1.0)	0.004	0.44
WV3 … have you remained silent despite having information that could have prevented a safety incident in the unit or service where you are training?	0.4 (0.8)	0.4 (0.8)	0.4 (0.9)	0.916	0.50	0.4 (0.7)	0.5 (0.8)	0.117	0.47
WV4 … have you avoided warning any professional in the unit or service where you are training that they were overlooking important patient safety rules/standards?	0.6 (0.9)	0.6 (0.9)	0.7 (1.0)	0.176	0.47	0.7 (1.0)	0.6 (0.9)	0.636	0.49
Total withholding voice^d^	5.1 (5.4)	4.9 (5.3)	5.6 (5.5)	0.065	0.46	4.8 (5.1)	5.4 (5.6)	0.235	0.47
**Speaking up** *(Cronbach’s α = 0.85; McDonald’s ω = 0.85)* *Over the last 4 weeks, how often…*	**Total** ^c^ **(*N* = 771)**	**Female (*n* = 565)**	**Male (*n* = 206)**	***p***	**PS**	**Nursing (*n* = 305)**	**Medicine (*n* = 374)**	***p***	**PS**
SU1 … have you explicitly shared your patient safety concerns with your supervisor, mentor, or other professionals on the unit or service?	1.7 (1.3)	1.7 (1.3)	1.8 (1.3)	0.130	0.47	1.9 (1.2)	1.6 (1.2)	0.001	0.43
SU2 … have you helped prevent another professional from making an error that could have caused harm to a patient?	1.3 (1.1)	1.2 (1.1)	1.4 (1.2)	0.010	0.44	1.4 (1.2)	1.2 (1.1)	0.003	0.44
SU3 … have you warned a professional in the unit or department where you are training that they were overlooking important patient safety rules/standards?	1.0 (1.1)	0.9 (1.0)	1.2 (1.1)	0.003	0.43	1.1 (1.1)	0.9 (1.0)	0.007	0.44
SU4 … have you prevented an incident from occurring by making concrete proposals to increase patient safety?	1.0 (1.1)	1.0 (1.0)	1.2 (1.0)	0.001	0.43	1.2 (1.1)	0.9 (1.0)	0.002	0.44
Total speaking up^d^	8.8 (6.5)	8.4 (6.4)	9.9 (6.8)	0.003	0.43	9.9 (6.6)	8.1 (6.3)	<0.001	0.42
**Perceived barriers** *(Cronbach’s α = 0.86; McDonald’s ω = 0.86)*	**Total** ^c^ **(*N* = 771)**	**Female (*n* = 565)**	**Male (*n* = 206)**	***p***	**PS**	**Nursing (*n* = 305)**	**Medicine (*n* = 374)**	***p***	**PS**
PB1. It is not clear that the situation represents a risk for a patient	0.9 (0.8)	0.9 (0.8)	0.9 (0.8)	0.512	0.49	0.8 (0.8)	0.9 (0.8)	0.406	0.48
PB2. Fear of a negative reaction from professionals or teachers/mentors	1.3 (1.1)	1.3 (1.0)	1.2 (1.1)	0.127	0.47	1.1 (1.0)	1.4 (1.1)	0.001	0.43
PB3. The presence of patients at that moment	1.1 (1.0)	1.2 (1.0)	1.0 (0.9)	0.060	0.46	1.0 (0.9)	1.2 (1.0)	<0.001	0.42
PB4. Doubts about how best to say it	1.2 (0.9)	1.2 (0.9)	1.1 (0.9)	0.035	0.45	1.1 (0.9)	1.2 (0.9)	0.025	0.45
PB5. Feel that one lacks sufficient social and communication skills to talk about it	0.9 (0.9)	1.0 (0.9)	0.8 (0.9)	0.002	0.43	0.8 (0.8)	1.0 (0.9)	0.009	0.45
PB6. The unpredictable reaction of the service or unit manager	1.1 (1.0)	1.2 (1.0)	1.0 (1.0)	0.057	0.46	1.0 (0.9)	1.2 (1.0)	0.003	0.44
PB7. Lack of self-confidence to discuss these issues with mentors or professionals	0.9 (0.9)	1.0 (0.9)	0.8 (0.9)	0.002	0.43	0.8 (0.8)	1.0 (1.0)	0.003	0.44
PB8. Believe that talking about these issues will negatively impact my current and future involvement with the centre	1.1 (1.0)	1.1 (1.0)	1.0 (1.0)	0.102	0.46	1.0 (0.9)	1.1 (1.0)	0.112	0.47
Total perceived barriers^d^	19.8 (12.6)	20.5 (12.6)	17.9 (12.4)	0.011	0.44	17.5 (11.7)	21.0 (12.7)	<0.001	0.42

^a^ For perceived concerns (PC), withholding voice (WV), speaking up (SU) categories were presented as: “never” (0 times in the last 4 weeks), “rarely” (1-2 times in the last 4 weeks), “sometimes” (3–5 times in the last 4 weeks), “often” (6–10 times in the last 4 weeks), and “very often” (more than 10 times in the last 4 weeks).

^b^ For PB, categories were presented as: “not at all” (0), “partially” (1), “moderately” (2), and “completely” (4).

^c^ Total column includes psychology students.

^d^ Ranges of total scores for the scales: perceived concerns (PC) (0–12), withholding voice (WV) (0–16), speaking up (SU) (0–16), PB (0–24).

**TABLE 4:** Mean (SD) responses to climate survey items (psychological safety for speaking up -PSS-, encouraging environment for speaking up -EES-, and resignation towards speaking up -RES) by sex and student profile^a^ (Colombia, Mexico, and Spain, 2021–2022).

**Psychological safety for speaking up** *(Cronbach’s α = 0.83; McDonald’s ω = 0.83)*	**Total^b^ (*N* = 771)**	**Female (*n* = 565)**	**Male (*n* = 206)**	***p***	**PS**	**Nursing (*n* = 305)**	**Medicine (*n* = 374)**	***p***	**PS**
PSS1. I can rely on my colleagues (other trainees) whenever I encounter difficulties in my work	5.4 (1.8)	5.4 (1.8)	5.3 (1.7)	0.179	0.47	5.3 (1.9)	5.4 (1.7)	0.974	0.50
PSS2. I can rely on my mentor whenever I encounter difficulties in my work as a trainee	5.6 (1.6)	5.7 (1.6)	5.5 (1.7)	0.475	0.48	5.7 (1.6)	5.5 (1.6)	0.003	0.44
PSS3. The culture (explicit and implicit norms and values) existing in the unit or service where I am training makes it easy to speak up about patient safety concerns	4.9 (1.9)	5.0 (1.9)	4.9 (1.9)	0.520	0.49	5.3 (1.7)	4.7 (1.9)	<0.001	0.40
PSS4. My colleagues (other trainees) react appropriately when I speak up about my patient safety concerns	5.3 (1.8)	5.4 (1.7)	5.1 (1.9)	0.042	0.45	5.3 (1.8)	5.2 (1.8)	0.292	0.48
PSS5. My professors or mentor react appropriately when I speak up about my patient safety concerns	5.3 (1.8)	5.3 (1.8)	5.3 (1.7)	0.508	0.49	5.5 (1.7)	5.1 (1.8)	<0.001	0.41
Total psychological safety for speaking up^c^	26.6 (6.8)	26.8 (6.7)	26.1 (7.1)	0.309	0.48	27.2 (7.0)	25.9 (6.6)	<0.001	0.42
**Encouraging Environment for Speaking up** *(Cronbach’s α = 0.86; McDonald’s ω = 0.87)*	**Total** ^b^ **(*N* = 771)**	**Female (*n* = 565)**	**Male (*n* = 206)**	***p***	**PS**	**Nursing (*n* = 305)**	**Medicine (*n* = 374)**	***p***	**PS**
EES1. In the unit or service where I am training, I notice that professionals naturally speak up about their patient safety concerns	4.9 (1.9)	4.8 (2.0)	4.9 (1.9)	0.778	0.49	4.9 (2.0)	4.9 (1.8)	0.464	0.48
EES2. Professionals in the service or unit where I am training encourage me to speak up about my patient safety concerns	4.6 (2.1)	4.6 (2.0)	4.5 (2.1)	0.862	0.50	4.8 (2.0)	4.4 (2.0)	0.002	0.43
EES3. My professors or mentor encourage me to speak up about my patient safety concerns	5.0 (1.9)	5.0 (1.9)	4.8 (2.0)	0.129	0.47	5.2 (1.8)	4.7 (1.9)	<0.001	0.40
Total encouraging environment for speaking up^c^	14.4 (5.2)	14.5 (5.2)	14.3 (5.3)	0.628	0.49	15.0 (5.3)	14.0 (5.0)	0.001	0.43
**Resignation towards Speaking up** ^d^	**Total** ^b^ **(*N* = 771)**	**Female (*n* = 565)**	**Male (*n* = 206)**	***p***	**PS**	**Nursing (*n* = 305)**	**Medicine (*n* = 374)**	***p***	**PS**
RES1. Suggesting changes to improve patient safety and no one listens to me is frustrating	3.3 (2.3)	3.3 (2.4)	3.4 (2.2)	0.833	0.50	3.4 (2.4)	3.3 (2.3)	0.702	0.49
RES2. I find it challenging to bring up my concerns about patient safety with professors and mentors	3.2 (2.1)	3.3 (2.2)	3.2 (2.1)	0.619	0.49	3.2 (2.2)	3.3 (2.1)	0.352	0.48
Total resignation towards speaking up^c^	6.6 (4.0)	6.6 (4.0)	6.5 (3.8)	0.838	0.50	6.6 (4.1)	6.7 (3.9)	0.741	0.49

^a^ For psychological safety for speaking up (PSS), encouraging environment for speaking up (EES) and resignation towards speaking up (RES) categories were presented as: “not applicable” (0), “strongly disagree” (1), “disagree” (2), “slightly disagree” (3), “neutral” (4), “slightly agree” (5), “agree” (6), and “strongly agree” (7).

^b^ Total column includes psychology students.

^c^ Ranges of total scores for the scales: psychological safety for speaking up (PSS) (0-35), encouraging environment for speaking up (EES) (0-21), resignation towards speaking up (RES) (0-14). Negatively worded items were reverse coded for the total score on the scales.

^d^ Reliability coefficients cannot be estimated because the number of items is less than 3.

### Differences Between Healthcare Students With and Without Practical Experience in Clinical or Social-Healthcare Settings

On both the behavioural and climate scales, students with practice (wp) scored significantly higher compared to those who had not yet undertaken practice in clinical or health and social care settings (w/op) (Supplementary Material S2).

In the 4 weeks before the survey, students with practice reported a higher frequency of perceived concerns about patient safety (wp M = 7.2, SD = 4.7 vs. w/op M = 5.5, SD = 4.4; *p* < 0.001, PS = 0.40) and of withholding voice behaviours (wp M = 5.1, SD = 5.4 vs. w/op M = 2.9, SD = 4.1; *p* < 0.001, PS = 0.36). This group also showed a higher attitude of resignation toward speaking up (wp M = 6.6, SD = 4.0 vs. w/op M = 4.8, SD = 4.4; *p* < 0.001, PS = 0.38). In contrast, no differences were observed in perceived barriers to openly discussing risks and issues affecting patient safety (wp M = 19.8, SD = 12.6 vs. w/op M = 19.6, SD = 13.8; *p* = 0.560), except the presence of patients (wp M = 1.1, SD = 1.0 vs. w/op M = 0.9, SD = 0.9; *p* = 0.003, PS = 0.45) and the lack of social and communication skills (wp M = 0.9, SD = 0.9 vs. w/op M = 1.1, SD = 1.0; *p* = 0.020, PS = 0.46).

Students with previous experience also reported a higher frequency of speaking up about patient safety behaviours during the 4 weeks before completing the questionnaire and the perception of a more encouraging and psychologically safe environment for speaking up.

In the responses to the vignette, no differences were observed according to experience in terms of the realism and riskiness of the situation described, nor in terms of the discomfort of asking the professional to follow safety rules (*p* > 0.05). However, students who had not been in practice reported a higher likelihood of warning the professional to sanitise their hands before caring for another patient (wp M = 5.4, SD = 1.7 vs. w/op M = 5.8, SD 1.5; *p* < 0.001, PS = 0.43).

### Safety Concerns, Barriers for Speaking Up, and Speaking Up Behaviours

Responses to the three behavioural scales (PC, WV, and SU) and perceived barriers (PB) to speaking up are showed in Table 2. In the experienced group, 88.3% of the students had been concerned about patient safety at least once during the previous 4 weeks. These concerns were accompanied by speaking up behaviours, i.e. 68.9% of respondents had prevented a colleague from making an error that could have harmed a patient. However, about four out of 10 students acknowledged that they had remained silent when witnessing a risky situation for patients. The most common barriers to speaking up about patient safety were, in order, fear of bad reactions from professionals or mentors, doubts about the best way to say it, uncertainty about the response of the unit manager, and the presence of patients when the risky practice is detected.

Table 3 shows the mean comparisons by sex (women vs. men) and student profile (nursing vs. medicine) on the behavioural scales and perceived barriers. Overall, men reported a higher frequency of patient safety concerns and speaking up behaviours compared to women. In contrast, women scored higher than men on perceived barriers to speaking up about patient safety, especially about internal factors (competence, skill, and self-confidence). However, the effect size of the observed differences was very small, with PS values between 0.42 and 0.50. No gender differences were observed in the frequency of withholding voice behaviours. In terms of student profile, doctors perceived more barriers than nurses to expressing patient safety concerns and initiatives, and 25.4% (*n* = 95) of doctors versus 16.7% (*n* = 51) of nurses reported having kept silent in situations of patient risk at least once during the last month (Chi-square = 14.233, df = 4; *p* = 0.007, Cramer’s V = 0.14). Consistently, nursing students reported a higher frequency of speaking up behaviours during the last 4 weeks compared to medical students.

### Speaking Up Related Climate

The responses to the climate items are shown in Table 4. Five hundred and twenty-nine (68.6%) students with clinical experience said they relied on their mentor when difficulties arose in their work as a trainee and 52.1% (*n* = 402) agreed that their professors or mentors encouraged them to speak up about their patient safety concerns. However, 17.5% (*n* = 135) acknowledged that bringing up such concerns with their supervisors was perceived as challenging. In terms of sex comparison, 65.9% (*n* = 372) of women versus 58.3% (*n* = 120) of men agreed that peers reacted well when expressing their patient safety concerns (Chi-square = 15.394, df = 7; *p* = 0.031, Cramer’s V = 0.14). No other differences were observed between men and women in perceived climate. Regarding differences by student profile, in general, nurses perceived a more encouraging and psychologically safe climate for speaking up compared to doctors. However, the effect size of these differences was very small, with PS values between 0.40 and 0.50.

### Evaluation of the Hand Hygiene Error Vignette

Table 5 shows the students’ assessments of the vignette. In general, students did not rate the hand hygiene error as a very realistic situation. However, they considered the situation to be quite dangerous for the patients. The speaking up behaviour was rated as probable, and the discomfort associated with risk communication as neutral. Men and women rated the situation similarly. Compared to physicians, nurses considered the vignette more realistic and reported a higher likelihood of speaking up despite feeling more uncomfortable, although the effect size of these differences was very small (PS values < 0.50).

**TABLE 5:** Mean (SD) vignette ratings by sex and student profile (Colombia, Mexico, and Spain, 2021–2022).

	Realistic	Risk of harm	Likelihood to speak up	Discomfort
Total	3.9 (1.9)	5.8 (1.4)	5.4 (1.7)	4.0 (1.9)
Sex				
Female	3.9 (1.9)	5.8 (1.4)	5.4 (1.7)	3.9 (1.9)
Male	3.7 (1.9)	5.7 (1.4)	5.3 (1.8)	4.1 (1.9)
*p*	0.192	0.166	0.661	0.261
PS	0.47	0.47	0.49	0.47
Student profile				
Nursing	4.2 (2.0)	5.7 (1.4)	5.6 (1.7)	4.3 (1.9)
Medicine	3.8 (1.8)	5.7 (1.3)	5.2 (1.7)	3.7 (1.9)
*p*	0.002	0.468	<0.001	<0.001
PS	0.43	0.48	0.42	0.42

All ratings measured on a seven-point scale. Realistic (1 = not at all, 7 = very realistic), Risk of harm (1 = not dangerous at all, 7 = extremely dangerous), Likelihood to speak up (1 = very unlikely, 7 = highly likely), and Discomfort (1 = not at all uncomfortable, 7 = extremely comfortable).

### Factors Associated With Speaking Up and Withholding Voice Behaviours

The results of the binary logistic regression with the frequency of speaking up and withholding voice as dependent variables are shown in Table 6. Perceived patient safety concerns were associated with speaking up and withholding voice behaviours (*p* < 0.001). Being male, a nursing student and reporting higher scores on the encouraging environment scale were also associated with a greater likelihood of frequently reporting speaking up (*p* < 0.01). Higher levels of resignation towards speaking up and perceived barriers were associated with higher frequencies of voice withholding (*p* < 0.01). In contrast, a higher level of psychological safety in expressing the behaviours was associated with a lower likelihood of reporting high frequencies of speaking up (*p* = 0.001).

**TABLE 6:** Results of binary logistic regression analysis with frequent speak up and frequent withholding voice as dependent variables (Colombia, Mexico, and Spain, 2021–2022).

Frequent speaking up	Or (95% CI)	*p*
Sex (0: male)	0.501 (0.328–0.765)	0.001
Age	1.008 (0.974–1.044)	0.632
Student profile (0: nursing)	0.606 (0.419–0.875)	0.008
Perceived concerns	1.282 (1.218–1.350)	<0.001
Perceived barriers	1.001 (0.985–1.018)	0.889
Psychological safety for speaking up	1.021 (0.985–1.058)	0.264
Encouraging environment for speaking up	1.082 (1.029–1.138)	0.002
Resignation towards speaking up	1.006 (0.959–1.054)	0.817
**Frequent withholding voice**	**OR (95% CI)**	***p***
Sex (0: male)	0.727 (0.483–1.095)	0.127
Age	1.031 (0.995–1.068)	0.088
Student profile (0: nursing)	0.952 (0.661–1.373)	0.794
Perceived concerns	1.190 (1.135–1.248)	<0.001
Perceived barriers	1.046 (1.028–1.064)	<0.001
Psychological safety for speaking up	0.959 (0.924–0.995)	0.026
Encouraging environment for speaking up	0.993 (0.943–1.045)	0.776
Resignation towards speaking up	1.074 (1.024–1.126)	0.003

## Discussion

This study shows that patient safety concerns and the observation of non-compliance with safety standards during the care process are frequent experiences among Ibero-American students of healthcare disciplines.

Of the participants in our study, nine out ten stated they had specific concerns about patient safety in the month before the survey. This finding is consistent with the experience reported by healthcare professionals in Switzerland [35], although somewhat higher than the frequency observed among medical students in Austria [27]. These similarities and differences held for speaking up behaviours when avoiding an error that could have harmed patients, with the only difference being that, in this case, the frequency was higher among Ibero-American students (68%) than among Austrian students (44%). This difference in speaking up frequency could be explained by the lower frequency with which Austrians experienced patient safety concerns.

Withholding voice behaviours were also relatively frequent among Ibero-American students. The figures were similar to those of Austrian medical students, although higher than those observed among health professionals [27, 35]. Our study showed that the main barriers to speaking up identified by Ibero-American students were social (reaction of others) and personal (lack of ability). Fear of damaging relationships as a result of this type of communication, the unpredictability of others’ reactions, the personality of senior staff (e.g., grumpy or stubborn), hierarchical and power differences, fear of punishment and the desire not to break unwritten rules or to preserve a good team climate are some of the most frequently reported reasons in clinical settings for withholding voice behaviours [27, 36–39].

In our study, men reported a higher frequency of safety concerns and speaking up behaviours compared to women. This finding may be related to women perceiving more barriers to expressing patient safety concerns. Along these lines, female students reported more frequently than their male peers that their misgivings about speaking up were associated with their lack of skill, denoting lower self-confidence. A similar result was obtained by Chen et al. (2023) [40] in a speaking up simulation course, where male medical students showed higher rates of speaking up than their female peers in life-threatening error scenarios. Analysing these sex differences from a gender perspective might suggest the existence of a bias in the treatment and attributed competence that women receive in clinical contexts versus that received by men [41, 42]. When analysed the data by the healthcare discipline, nursing students reported a higher frequency of speaking up behaviours compared to physicians who perceived more barriers to expressing patient safety concerns and initiatives. No differences were found in the frequency of concerns and withholding voice behaviours by student profile.

Regarding perceived climate, Ibero-American students showed intermediate average scores, suggesting that the working environment was neither perceived as extremely favourable nor unfavourable for speaking up. However, ratings were slightly more positive on the scales of psychological safety and encouraging environment for speaking up as opposed to resignation, suggesting a slightly favourable perception of the working environment. The analysis results by student profile are consistent with findings in samples of professionals, where nursing reported a more positive and encouraging psychological safety climate for speaking up compared to medicine. Differences between nursing and medicine in aspects related to patient safety, although not universal, are observed with some frequency [15, 17].

The logistic regression results were in line with what has been observed in other studies, as perceived worries were strongly associated with speaking up and withholding voice. These two voice behaviours, although antagonistic in their direction, are common and coexist. Also, being a nurse and perceiving an encouraging environment were more strongly associated with openly expressing concerns [35]. Other motivating factors that seem to explain speaking up behaviours are the existence of a positive (non-judgemental, non-punitive) safety culture, supportive unit manager and role models, positive reactions from others, familiarity with team members, high-risk situations for patients and staff and some personal characteristics and beliefs (assertiveness, confidence, etc.) [40].

As expected, students with experience in clinical settings scored higher than those without experience on all scales of the questionnaire, both those that are positively related to speaking up and those that inhibit it. This aspect is explained by the fact that practicing students have direct exposure to care activities, actual patients, cultural and climate factors, and the working dynamics of a particular healthcare institution and are assumed to have a greater awareness of risks, while the perception of students who have not yet undertaken practice is mediated by a more indirect, theoretical experience and developed under controlled learning conditions. This result is like the one found in other studies comparing students in their first and last years [27]. Along these lines, our untrained students reported a higher likelihood of warning the practitioner to sanitise their hands when analysing the error vignette. This possible overestimation of their willingness to speak up could be due to the difficulty in realistically analysing the situation. In contrast, trainees with practice are more likely to approach this exercise with a specific personalisation of the actors and the context.

### Strengths and Limitations

Our study is the first to analyse speaking up behaviours in students of health disciplines in Ibero-America. To this end, a version of the SUPS-Q has been successfully adapted to Spanish. These results provide insights for planning actions to foster safe clinical environments for patients and the learning of future healthcare professionals. However, our study also has significant limitations. The main objective of this study was to explore speaking up behaviours, barriers to openly expressing patient safety concerns, and perceived psychological safety climate in Ibero-American healthcare trainees, and not the validation of the measurement instrument. However, it was necessary to analyse the face and construct validity of the questionnaire when first translated into Spanish to verify whether the data confirmed the original factor structure. Future studies should address the analysis of the psychometric properties of the SUPS-Q in its Spanish version, including the determination of discriminant, convergent and criterion validity. The sample size may have had a paradoxical effect on the results regarding generalisability and significance. The small sample size relative to the study population and possible response bias limited the results’ generalisability. Conversely, the large sample size in absolute terms may have been behind the statistically significant differences observed, which would explain the small effect size values obtained. Responses may have been affected by social desirability and recall biases as the questionnaire included retrospective questions. The study period coincided with the COVID-19 pandemic, so many students had their opportunities for clinical internships restricted or postponed. Also, the cross-sectional study design did not allow for establishing cause-effect relationships between the variables of interest. The international nature of the study, although valuable in its scope, adds limitations associated with differences between countries regarding curricula, timing of clinical practices, cultural factors, and the nature of healthcare systems. However, intra-institutional variability between units is sometimes equally high. The decision not to conduct cross-country analyses was based on the above reasons, along with the impossibility of determining the cultural invariance of the instrument and the commitment to researchers in the different participating countries to adopt a joint learning approach rather than a comparison or stratification focus. The need to progress in patient safety and psychological safety is still a common challenge for most countries. Finally, psychology students were excluded from the comparative analyses by discipline. However, this decision was made considering Schwappach and Niederhauser’s (2019) [43] findings that psychologists’ responses systematically deviated from other professional groups due to their lower exposure to errors or non-compliance with safety rules and familiarity with clinical standards of care.

### Implications for Practice and Future Research

Patient safety concerns and observation of risky practices in clinical settings are opportunities to learn about and provide safer care for patients. Ibero-American students experience such situations with some frequency, but they are not always speaking up about their concerns. It is a challenge to create clinical settings that facilitate the open expression of these concerns, even more so among students, despite evidence that they can be a valid information source for improving patient safety [44].

We have not found interventions to encourage speaking up among students specifically developed in Latin America. However, in other regions, several educational programmes have been developed that have shown good results in mitigating barriers to speaking up and improving attitudes towards voicing opinions in healthcare teams [45, 46]. Other timely measures include involving preceptors in creating safe clinical learning environments [47] and appointing trusted role models to advise and support students in raising a concern along the lines of the Freedom To Speak Up Guardian and Confidential Contacts in the United Kingdom National Health Service [48]. Interestingly, initiatives have begun emerging that recognise the interdependence of sender and receiver roles in speaking up behaviours and consequently train healthcare professionals in speaking up skills and responding strategies [49]. This training should be extended to mentors, middle managers, and managers in healthcare institutions to mitigate the hierarchical barrier.

Future research should explore the experience of Ibero-American students as recipients of speaking up behaviours. Also, speaking up training in the early stages of training promotes higher commitment to patient safety in later practice in the healthcare profession. When designing interventions, differences by gender and professional profile should be considered. Research is also needed to measure the impact of speaking up initiatives on patient-level safety outcomes.

### Conclusion

This study revealed that Ibero-American students in healthcare disciplines often experience patient safety concerns that they need to express openly to peers or superiors. However, this communication is often constrained by several personal and cultural barriers present in clinical settings. These findings suggest the need for action to train students in communication and teamwork skills that support confidence for speaking up and to create safe spaces for patients and professionals. The results also encourage ongoing learning and continuous improvement challenges in healthcare institutions with a greater focus on interprofessional and intergenerational work.
