# Supplementary material for: Speaking Up About Patient Safety, Withholding Voice and Safety Climate in Clinical Settings: a Cross-Sectional Study Among Ibero-American Healthcare Students
Source: Int J Public Health. 2024 Jul 1;69:1607406. doi: 10.3389/ijph.2024.1607406 (PMC11246871; doi:10.3389/ijph.2024.1607406)
Supplement: Supplementary file 1 [file DataSheet2.pdf]

Supplementary file 2. Descriptive analyses of SUPS-Q scales and Mann-Whitney U by students' level of practice (with vs. without practice)

Table 1. Mean (SD) responses to perceived concerns (PC), withholding voice (WV), speaking up behaviours (SU) and perceived barriers (PB) to speaking up by students' level of practice[a,b] (Colombia, Mexico, and Spain, 2021-2022)

Perceived concerns<br>*Over the last four weeks, how often…*	Total<br>(N = 1,152)	Without practice<br>(n = 381)	With practice<br>(n = 771)	p	PS
PC1 … have you had specific concerns about patient safety?	1.8 (1.2)	1.7 (1.3)	1.9 (1.1)	0.004	0.45
PC2 ... have you observed a failure/error that, if uncaptured timely, could be harmful to patients?	0.9 (1.0)	0.7 (0.9)	1.0 (1.0)	<0.001	0.41
PC3 ... have you noticed that a professional in the unit or service in which you are training has not followed important patient safety rules or standards?	1.0 (1.1)	0.7 (0.9)	1.1 (1.2)	<0.001	0.39
Total perceived concerns[c]	6.6 (4.7)	5.5 (4.4)	7.2 (4.7)	<0.001	0.40
**Withholding voice**<br>*Over the last four weeks, how often…*	**Total<br>(N = 1,152)**	**Without practice<br>(n = 381)**	**With practice<br>(n = 771)**	**p**	**PS**
WV1 … have you kept ideas that could improve patient safety in the unit or department where you are training?	1.0 (1.1)	0.7 (1.0)	1.2 (1.2)	<0.001	0.39
WV2 ... have you chosen to remain silent and not say anything when witnessing a risky situation for a patient?	0.5 (0.9)	0.3 (0.6)	0.6 (1.0)	<0.001	0.41
WV3 ... have you remained silent despite having information that could have prevented a safety incident in the unit or service where you are training?	0.4 (0.8)	0.3 (0.6)	0.4 (0.8)	<0.001	0.45
WV4 … have you avoided warning any professional in the unit or service where you are training that they were overlooking important patient safety rules/standards?	0.5 (0.9)	0.3 (0,7)	0.6 (0.9)	<0.001	0.41
Total withholding voice[c]	4.3 (5.1)	2.9 (4.1)	5.1 (5.4)	<0.001	0.36
**Speaking up**<br>*Over the last four weeks, how often…*	**Total<br>(N = 1,152)**	**Without practice<br>(n = 381)**	**With practice<br>(n = 771)**	**p**	**PS**
SU1 … have you explicitly shared your patient safety concerns with your supervisor, mentor, or other professionals on the unit or service?	1.5 (1.3)	1.0 (1.2)	1.7 (1.3)	<0.001	0.34
SU2 ... have you helped prevent another professional from making an error that could have caused harm to a patient?	1.1 (1.1)	0.7 (1.0)	1.3 (1.1)	<0.001	0.35
SU3 ... have you warned a professional in the unit or department where you are training that they were overlooking important patient safety rules/standards?	0.9 (1.0)	0.7 (1.0)	1.0 (1.1)	<0.001	0.40
SU4 … have you prevented an incident from occurring by making concrete proposals to increase patient safety?	0.9 (1.0)	0.6 (1.0)	1.0 (1.1)	<0.001	0.38
Total speaking up[c]	7.6 (6.6)	5.3 (6.1)	8.8 (6.5)	<0.001	0.33

Perceived barriers	Total (N = 1,152)	Without practice (n = 381)	With practice (n = 771)	P	PS
PB1. It is not clear that the situation represents a risk for a patient.	0.9 (0.8)	0.9 (0.9)	0.9 (0.8)	0.985	0.50
PB2. Fear of a negative reaction from professionals or teachers/mentors.	1.2 (1.1)	1.2 (1.1)	1.3 (1.1)	0.420	0.49
PB3. The presence of patients at that moment.	1.1 (1.0)	0.9 (0.9)	1.1 (1.0)	0.003	0.45
PB4. Doubts about how best to say it.	1.2 (0.9)	1.1 (0.9)	1.2 (0.9)	0.391	0.49
PB5. Feel that one lacks sufficient social and communication skills to talk about it.	1.0 (0.9)	1.1 (1.0)	0.9 (0.9)	0.020	0.46
PB6. The unpredictable reaction of the service or unit manager.	1.1 (1.0)	1.1 (1.0)	1.1 (1.0)	0.649	0.49
PB7. Lack of self-confidence to discuss these issues with mentors or professionals.	0.9 (0.9)	1.0 (1.0)	0.9 (0.9)	0.118	0.47
PB8. Believe that talking about these issues will negatively impact my current and future involvement with the centre.	1.1 (1.0)	1.0 (1.0)	1.1 (1.0)	0.294	0.48
Total perceived barriers[c]	19.7 (13.0)	19.6 (13.8)	19.8 (12.6)	0.560	0.49

[a]For perceived concerns (PC), withholding voice (WV) and speaking up (SU) categories were presented as: “never” (0 times in the last 4 weeks), “rarely” (1-2 times in the last 4 weeks), “sometimes” (3-5 times in the last 4 weeks), “often” (6-10 times in the last 4 weeks), and “very often” (more than 10 times in the last 4 weeks)

[b]For perceived barriers (PB) category were presented as: “not at all” (0), “partially” (1), “moderately” (2), and “completely” (4)

[c]Ranges of total scores for the scales: perceived concerns -PC- (0-12), withholding voice -WV- (0-16), speaking up -SU- (0-16), and perceived barriers -PB- (0-24)

Table 2. Mean (SD) responses to climate survey items (psychological safety for speaking up -PSS-, encouraging environment for speaking up -EES-, and resignation towards speaking up -RES) by students' level of practice[a] (Colombia, Mexico, and Spain, 2021-2022)

Psychological Safety for Speaking up	Total (N = 1,152)	Without practice (n = 381)	With practice (n = 771)	p	PS
PSS1. I can rely on my colleagues (other trainees) whenever I encounter difficulties in my work.	5.0 (2.2)	4.1 (2.7)	5.4 (1.8)	<0.001	0.37
PSS2. I can rely on my mentor whenever I encounter difficulties in my work as a trainee.	5.2 (2.1)	4.2 (2.7)	5.6 (1.6)	<0.001	0.35
PSS3. The culture (explicit and implicit norms and values) existing in the unit or service where I am training makes it easy to speak up about patient safety concerns.	4.5 (2.3)	3.6 (2.7)	4.9 (1.9)	<0.001	0.36
PSS4. My colleagues (other trainees) react appropriately when I speak up about my patient safety concerns.	4.8 (2.2)	3.9 (2.7)	5.3 (1.8)	<0.001	0.36
PSS5. My professors or mentor react appropriately when I speak up about my patient safety concerns.	4.9 (2.2)	4.0 (2.7)	5.3 (1.8)	<0.001	0.36
Total psychological safety for speaking up[b]	24.4 (9.5)	19.9 (12.2)	26.6 (6.8)	<0.001	0.35
**Encouraging Environment for Speaking up**	**Total (N = 1,152)**	**Without practice (n = 381)**	**With practice (n = 771)**	**p**	**PS**
EES1. In the unit or service where I am training, I notice that professionals naturally speak up about their patient safety concerns.	4.3 (2.4)	3.3 (2.8)	4.9 (1.9)	<0.001	0.34
EES2. Professionals in the service or unit where I am training encourage me to speak up about my patient safety concerns.	4.1 (2.4)	3.2 (2.7)	4.6 (2.1)	<0.001	0.36
EES3. My professors or mentor encourage me to speak up about my patient safety concerns.	4.6 (2.3)	3.8 (2.7)	5.0 (1.9)	<0.001	0.38
Total encouraging environment for speaking up[b]	13.0 (6.4)	10.3 (7.5)	14.4 (5.2)	<0.001	0.35
**Resignation towards Speaking up**	**Total (N = 1,152)**	**Without practice (n = 381)**	**With practice (n = 771)**	**p**	**PS**
RES1. Suggesting changes to improve patient safety and no one listens to me is frustrating.	3.0 (2.4)	2.4 (2.5)	3.3 (2.3)	<0.001	0.38
RES2. I find it challenging to bring up my concerns about patient safety with professors and mentors.	2.9 (2.2)	2.4 (2.3)	3.2 (2.1)	<0.001	0.38
Total resignation towards speaking up[b]	6.0 (4.2)	4.8 (4.4)	6.6 (4.0)	<0.001	0.38

[a]For psychological safety for speaking up (PSS), encouraging environment for speaking up (EES) and resignation towards speaking up (RES) categories were presented as: "not applicable" (0), "strongly disagree" (1), "disagree" (2), "slightly disagree" (3), "neutral" (4), "slightly agree" (5), "agree" (6), and "strongly agree" (7)

[b]Ranges of total scores for the scales: psychological safety for speaking up -PSS- (0-35), encouraging environment for speaking up -EES- (0-21) and resignation towards speaking up -RES- (0-14). Negatively worded items were reverse coded for the total score on the scales

Table 3. Mean (SD) vignette ratings by students' level of practice (N = 1,152) (Colombia, Mexico, and Spain, 2021-2022)

	Realistic	Risk of harm	Likelihood to speak up	Discomfort
Total	3.9 (1.8)	5.8 (1.4)	5.5 (1.7)	4.0 (1.8)
Students' level of practice				
Without practice	3.9 (1.8)	5.7 (1.5)	5.8 (1.5)	4.0 (1.7)
With practice	3.9 (1.9)	5.8 (1.4)	5.4 (1.7)	4.0 (1.9)
p	0.600	0.668	<0.001	0.363
PS	0.49	0.49	0.43	0.48

All ratings measured on a seven-point scale. Realistic (1 = not at all, 7 = very realistic), Risk of harm (1 = not dangerous at all, 7 = extremely dangerous), Likelihood to speak up (1 = very unlikely, 7 = highly likely), and Discomfort (1 = not at all uncomfortable, 7 = extremely comfortable)
