# Supplementary material for: Speaking Up About Patient Safety, Withholding Voice and Safety Climate in Clinical Settings: a Cross-Sectional Study Among Ibero-American Healthcare Students
Source: Int J Public Health. 2024 Jul 1;69:1607406. doi: 10.3389/ijph.2024.1607406 (PMC11246871; doi:10.3389/ijph.2024.1607406)

Supplementary file 1. Structural Equation Model and standardized coefficients for the seven-factor model of SUPS-Q in Ibero-American healthcare students (Colombia, Mexico, and Spain, 2021-2022)

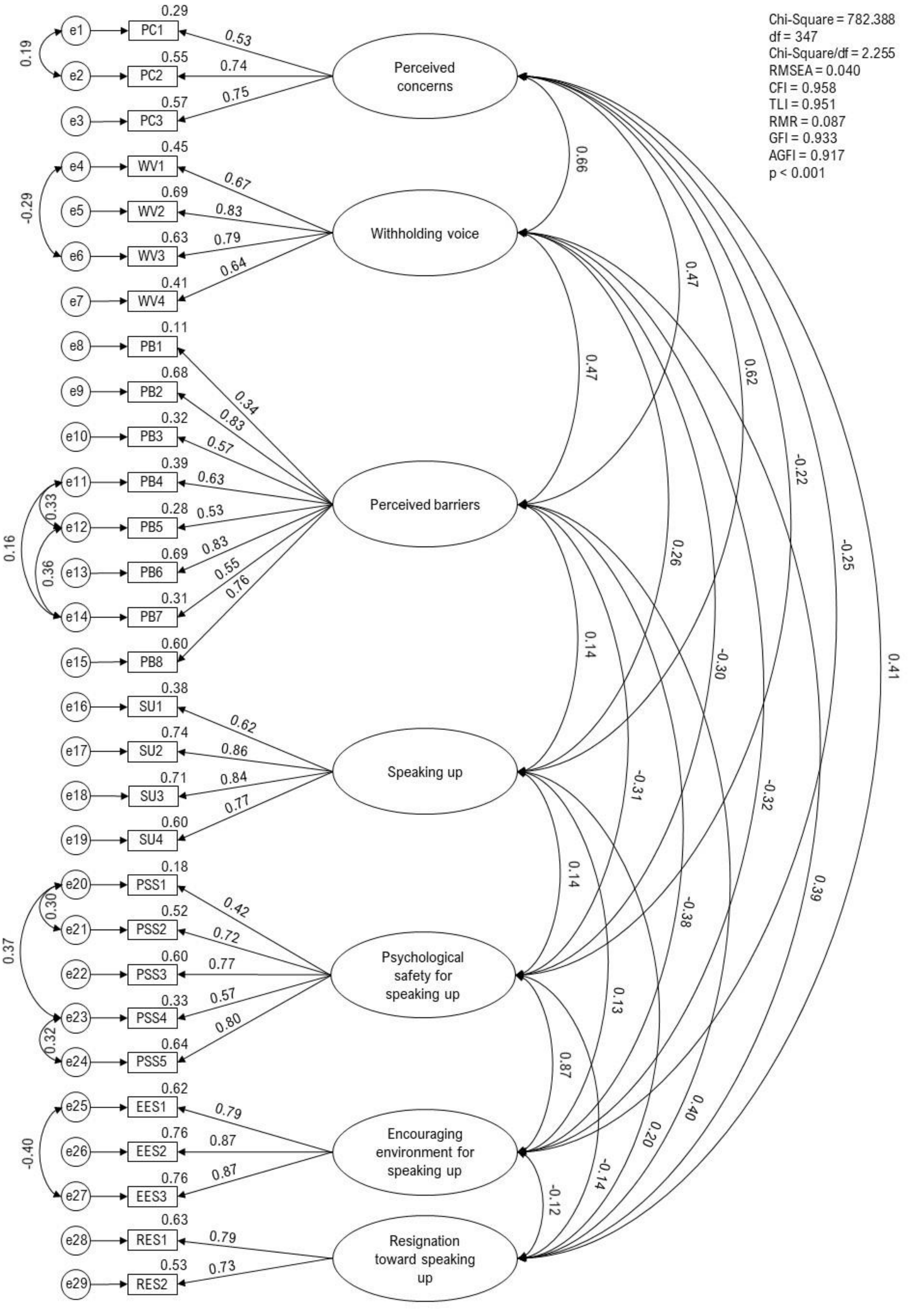

Supplement: Supplementary file 2 [file DataSheet1.pdf]
